# A Practical Guide for the Systemic Treatment of Biliary Tract Cancer in Canada

**DOI:** 10.3390/curroncol30080517

**Published:** 2023-07-25

**Authors:** Ravi Ramjeesingh, Prosanto Chaudhury, Vincent C. Tam, David Roberge, Howard J. Lim, Jennifer J. Knox, Jamil Asselah, Sarah Doucette, Nirlep Chhiber, Rachel Goodwin

**Affiliations:** 1 Division of Medical Oncology, Department of Medicine, Nova Scotia Health, Dalhousie University, Halifax, NS B3H 2Y9, Canada; 2 Department of Surgery and Oncology, McGill University Health Centre, Royal Victoria Hospital, Montreal, QC H4A 3J1, Canada; 3 Division of Medical Oncology, Department of Oncology, University of Calgary, Calgary, AB T2N 4N2, Canada; 4 Department of Radiology, Radiation Oncology and Nuclear Medicine, University of Montreal, Montreal, QC H3T 1A4, Canada; 5 Division of Medical Oncology, BC Cancer, Vancouver, BC V5Z 4E6, Canada; 6 Division of Medical Oncology and Hematology, Department of Medicine, Princess Margaret Cancer Centre, University of Toronto, Toronto, ON M5G 2M9, Canada; 7 Department of Medicine, Division of Medical Oncology, McGill University Health Centre, Montreal, QC H4A 3J1, Canada; 8 IMPACT Medicom Inc., Toronto, ON M6S 3K2, Canada; sarah@impactmedicom.com (S.D.);; 9 Division of Medical Oncology, The Ottawa Hospital Cancer Centre, University of Ottawa, Ottawa, ON K1H 8L6, Canada

**Keywords:** biliary tract cancer, cholangiocarcinoma, gallbladder carcinoma, systemic therapy, adjuvant therapy, neoadjuvant therapy, immunotherapy, targeted therapy

## Abstract

Biliary tract cancers (BTC) are rare and aggressive tumors with poor prognosis. Radical surgery offers the best chance for cure; however, most patients present with unresectable disease, and among those receiving curative-intent surgery, recurrence rates remain high. While other locoregional therapies for unresectable disease may be considered, only select patients may be eligible. Consequently, systemic therapy plays a significant role in the treatment of BTC. In the adjuvant setting, capecitabine is recommended following curative-intent resection. In the neoadjuvant setting, systemic therapy has mostly been explored for downstaging in borderline resectable tumours, although evidence for its routine use is lacking. For advanced unresectable or metastatic disease, gemcitabine-cisplatin plus durvalumab has become the standard of care, while the addition of pembrolizumab to gemcitabine-cisplatin has also recently demonstrated improved survival compared to chemotherapy alone. Following progression on gemcitabine-cisplatin, several chemotherapy combinations and biomarker-driven targeted agents have been explored. However, the optimum regimen remains unclear, and access to targeted agents remains challenging in Canada. Overall, this article serves as a practical guide for the systemic treatment of BTC in Canada, providing valuable insights into the current and future treatment landscape for this challenging disease.

## 1. Introduction

**Figure 1:**
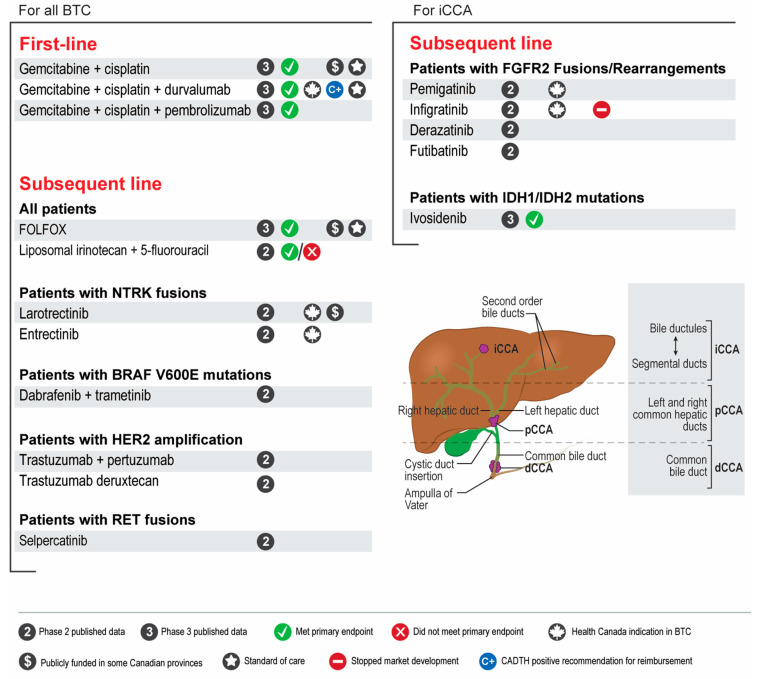
Summary of systemic treatment landscape for advanced unresectable or metastatic biliary tract cancers in Canada. BTC, biliary tract cancer; CADTH, Canadian Agency for Drugs and Technology in Health; iCCA, intrahepatic cholangiocarcinoma; dCCA, distal cholangiocarcinoma; pCCA, perihilar cholangiocarcinoma; FOLFOX, folinic acid, fluorouracil, oxaliplatin.

Biliary tract cancers (BTCs) are a group of neoplasms that are classified by location of origin within the biliary tract. This includes intrahepatic cholangiocarcinoma (iCCA), originating within the liver above the second-order bile ducts, extrahepatic cholangiocarcinoma (eCCA), located within the hepatoduodenal ligament, gallbladder carcinoma (GBC), originating in the gallbladder, and ampullary cancer (AC), originating in the ampulla of Vater (Figure 1). Extrahepatic CCA can be further divided into perihilar and distal disease; the former is located between the insertion of the cystic duct and the second-order bile ducts, and the latter originates in the common bile duct [1]. BTC subtypes have both distinct and overlapping features in disease presentation, dissemination, molecular profile, and natural history, which has implications on treatment options and adds to the complexity of managing this heterogenous disease [2].

The true incidence of BTC is unclear since some subtypes have been historically grouped with liver cancer in national databases (particularly iCCA) and few epidemiological studies have been published [3]. Estimates of the incidence of BTC differ by region, with age-standardized rates between 1 and 4 cases per 100,000 person-years for most regions in North America (including Canada) and Europe, while higher rates have been reported for countries in the Asia-Pacific region and South America [3]. Studies have also reported differences in incidence by subtype, with GBC having a higher incidence in females, and other BTC subtypes having a higher incidence in males [4,5,6,7]. Several studies have reported a rise in the incidence of iCCA over the last few decades, although it is unclear whether this increase is related to better diagnosis and classification of the disease in cancer databases, a higher prevalence of risk factors associated with iCCA, or a combination of factors [8,9]. Overall, as BTC is a relatively rare group of cancers (<0.5% of all cancer diagnoses in Canada per year) [10], this poses a challenge to conducting adequately powered, randomized trials that can provide evidence to guide treatment.

Biliary tract cancer is associated with a poor prognosis owing to its aggressive behaviour and late symptom presentation that results in a significant number of patients being diagnosed with advanced disease. Five-year survival rates range from 9–35% depending on primary site, with the worst survival reported in patients with iCCA and the best survival reported in patients with AC [6,11,12,13,14,15]. Distant metastatic disease at diagnosis is associated with a 5-year survival rate as low as 2–3% [12,14,15].

Radical surgery followed by adjuvant therapy offers the best chance of cure and should be the first treatment to be considered in consultation with a multidisciplinary team, including a hepatobiliary surgeon. Unfortunately, surgery is not indicated or feasible in over 60% of BTC patients at diagnosis for a variety of reasons, including presence of distant metastasis, vascular invasion not amenable to reconstruction, bilobar involvement, inability to reconstruct bile duct, insufficient volume or function of liver remnant, poor performance status, and liver cirrhosis [6,16]. Even among those receiving curative-intent surgery, rates of recurrence can range between 55–75% [17,18,19,20]. Intrahepatic CCA is associated with the highest risk of recurrence following curative surgery, with 5-year recurrence-free survival rates ranging from 2–39% [21].

Neoadjuvant chemoradiation with subsequent liver transplantation is a potential option for patients with liver-confined CCA based on data from small, single-center retrospective studies [22,23,24,25]. However, strict eligibility criteria, low availability of livers to transplant, and variability of results between studies limit its use. Other locoregional therapies such as radiofrequency ablation, chemoembolization, radioembolization, chemotherapy hepatic arterial infusion, external beam radiotherapy, and stereotactic body radiation therapy have also demonstrated efficacy in unresectable BTC, mostly in retrospective studies [26]. Despite advances in local therapies, high recurrence rates and a failure to meet eligibility criteria mean that systemic therapy continues to play a large role in the treatment of BTC. This document, developed by a pan-Canadian panel of medical, surgical, and radiation oncologists, provides guidance on the use of systemic therapy in patients with BTC in the setting of unresectable advanced disease, adjuvant therapy, and neoadjuvant therapy.

## 2. Systemic Therapy for Unresectable Advanced or Metastatic Biliary Tract Cancer

### 2.1. Recommendations

1. Patients with advanced unresectable or metastatic BTC should be considered for first-line treatment with gemcitabine-cisplatin plus immunotherapy (durvalumab).
2. Genomic profiling of relevant BTC genes by next-generation sequencing is strongly suggested for all patients with advanced unresectable or metastatic BTC that are fit to receive systemic therapy. Profiling is preferred at diagnosis to allow for treatment planning and access to targeted agents in the second line.
3. FOLFOX should be considered in the second line setting after progressing on gemcitabine-cisplatin-based therapy for patients with advanced BTC with no actionable genomic alterations.
4. Patients with CCA who harbour FGFR2 fusions should be considered for treatment with FGFR2 inhibitors (pemigatinib) after progressing on one prior line of systemic therapy.
5. Patients with CCA who harbour IDH1 mutations do not have access to IDH1 inhibitors in Canada. Alternative means of access may be considered but is challenging.
6. Patients with BTC who harbour NTRK fusions should be considered for treatment with NTRK inhibitors (entrectinib or larotrectinib) after progressing on one prior line of systemic therapy.
7. Patients with BTC who harbour other actionable genomic alterations (e.g., BRAF, HER2, RET, MSI) should be considered for targeted therapy through clinical trials or other means of access.
8. Locoregional therapies for palliation should be considered and discussed with multidisciplinary teams.

### 2.2. Discussion on First-Line Systemic Therapies

Prior to 2000, gemcitabine monotherapy was the standard therapy for the first-line treatment of advanced unresectable BTC, based on results from a clinical trial of pooled patients with pancreatic and biliary tract cancers [27]. In 2010, the addition of cisplatin to gemcitabine replaced this standard of care after it showed a significant reduction in the risk of death over gemcitabine alone in the phase III ABC-02 trial, specifically in patients with advanced or metastatic BTC (median overall survival [OS] 11.7 months vs. 8.1 months; HR, 0.64; 95% CI, 0.52–0.80; *p* < 0.001) [28] (Table 1 and Figure 1). In this trial, 410 patients were randomized to receive cisplatin 25 mg/m^2^ and gemcitabine 1000 mg/m^2^ administered on days 1 and 8 for eight 3-week cycles or gemcitabine 1000 mg/m^2^ alone on days 1, 8, and 15, up to six 4-week cycles. Although the ABC-02 trial stopped gemcitabine-cisplatin after eight cycles, many oncologists in Canada will discuss with patients the potential to continue dual chemotherapy or gemcitabine monotherapy until progression or dose-limiting toxicity [29]. This is based on practices from other tumour types which have demonstrated the benefit of continued palliative chemotherapy until progression [30]. The benefit of gemcitabine-cisplatin therapy beyond eight cycles in BTC is unclear, with some studies reporting conflicting results on the added value of continuous therapy [31,32]. Decisions to administer continuous chemotherapy should thus be made at the discretion of the physician, considering an individual patient’s tolerability, response to initial therapy, and preferences [33].

Other doublet chemotherapy regimens have since been compared against gemcitabine-cisplatin for the first-line treatment of BTC, none of which demonstrated a statistically significant improvement in OS (Table 1). Although gemcitabine-cisplatin has remained the standard chemotherapy backbone for BTC, gemcitabine alone or gemcitabine-oxaliplatin can be considered for patients who are anticipated to have poor tolerability to gemcitabine-cisplatin [33].

Several chemotherapy-based triplet regimens have been investigated in randomized trials with gemcitabine-cisplatin as the control arm; however, these regimens did not improve survival outcomes and were associated with increased toxicity (Table 1). These include modified FOLFIRINOX (fluorouracil, irinotecan, and oxaliplatin), gemcitabine-cisplatin plus albumin-bound paclitaxel, and gemcitabine-oxaliplatin-capecitabine [35,37,38]. The intensification of therapy with targeted agents against the vascular endothelial growth factor receptor have also failed to demonstrate a statistically significant improvement in PFS compared to gemcitabine-cisplatin alone [42,43].

Triplet therapy combining gemcitabine, cisplatin, and the anti-programed death-ligand 1 (PD-L1) checkpoint inhibitor durvalumab has demonstrated the first major improvement in frontline treatment for BTC in over a decade. This was demonstrated in the TOPAZ-1 study, a phase III, randomized, double-blind trial which evaluated gemcitabine-cisplatin plus durvalumab versus gemcitabine-cisplatin in 685 patients with advanced BTC [40] (Table 1). In this study, gemcitabine-cisplatin was given for up to eight cycles in both arms. In an updated analysis presented at the European Society for Medical Oncology (ESMO) 2022 annual conference, durvalumab plus gemcitabine-cisplatin led to a prolonged median OS (12.9 vs. 11.3 months) and a 24% reduction in the risk of death compared to gemcitabine-cisplatin plus placebo (HR 0.76; 95% CI 0.64–0.91) [39]. The estimated OS rate at 24 months was also significantly higher for the durvalumab versus placebo arm (23.6% vs. 11.5%). Compared with the control arm, the triplet therapy led to statistically significant improvements in PFS (median 7.2 vs. 5.7; HR 0.75; 95% CI 0.63–0.89; *p* = 0.001) and a higher overall response rate (26.7% vs. 18.7%) [40]. Rates of grade 3/4 adverse events and quality of life remained similar in both arms [40,44]. Results from the TOPAZ-1 trial have led to a change in international practice guidelines, including the National Comprehensive Cancer Network and ESMO guidelines, which now recommend durvalumab plus gemcitabine-cisplatin as a preferred standard of care treatment option for patients with advanced unresectable and metastatic BTC [33,45] (Figure 1).

The double-blind, placebo-controlled phase III KEYNOTE-966 trial also recently reported improved outcomes with the anti-programmed death-1 checkpoint inhibitor pembrolizumab plus gemcitabine-cisplatin compared to placebo plus gemcitabine-cisplatin [41]. In this study enrolling 1069 patients with advanced or metastatic BTC, gemcitabine could be administered beyond eight cycles in both treatment arms. This occurred in 43% of patients in the pembrolizumab group and 39% of patients in the placebo group, reflecting differences in practice across regions. Median OS was significantly prolonged in the pembrolizumab group compared to the placebo group (12.7 months vs. 10.9 months; *p* = 0.0034), and pembrolizumab plus gemcitabine-cisplatin was associated with a 17% reduction in the risk of death (HR 0.83; 95% CI 0.72–0.95). The estimated 24-month OS rates in the pembrolizumab and placebo arm were 25% and 18%, respectively. The median PFS was 6.5 months in the pembrolizumab arm and 5.6 months in the placebo arm (HR 0.86; 95% CI 0.75–1.00; *p* = 0.023); however, this improvement was not considered statistically significant given the applied significance boundary of *p* = 0.0125. The overall response rate (ORR, 29% in both arms) and treatment-related adverse events were similar in both arms.

Together, the TOPAZ-1 and KEYNOTE-966 trials confirm the benefit of adding immunotherapy to gemcitabine-cisplatin in patients with advanced unresectable or metastatic BTC. Although the TOPAZ-1 trial demonstrated a larger 2-year OS benefit for the durvalumab regimen over the control arm than what was reported for pembrolizumab in the KEYNOTE-966 study (12.1% vs. 7% improvement in 2-year OS), the allowance of continuous gemcitabine following eight completed cycles of chemotherapy in the KEYNOTE-966 study may have impacted the magnitude of benefit. Without direct comparison trials, the selection of immunotherapy is based on accessibility. Currently, only durvalumab has a Health Canada indication for locally advanced or metastatic BTC in combination with gemcitabine-based therapy.

### 2.3. Discussion on Subsequent-Line Systemic Therapies

Patients who have good performance status and experience disease progression following first-line chemotherapy may benefit from second-line therapy. However, a minority of patients (15–25%) are fit enough for this option [46]. The most common second-line treatment option in Canada for patients with BTC who have progressed on gemcitabine-cisplatin is the combination of fluorouracil, oxaliplatin, and leucovorin (FOLFOX) (Figure 1). This is based on the phase III ABC-06 study which met its primary endpoint demonstrating improved survival for the modified FOLFOX versus the active symptom control arms (median OS: 6.2 months vs. 5.3 months; HR 0.69; 95% CI 0.50–0.97; *p* = 0.031) [47]. Liposomal irinotecan plus 5-fluorouracil/leucovorin has also demonstrated efficacy in patients who have progressed on first-line gemcitabine-cisplatin. In the phase II NIFTY trial, a significantly longer median OS with liposomal irinotecan and 5-fluorouracil/leucovorin versus 5-fluorouracil/leucovorin alone was reported in this population of BTC patients (8.6 months vs. 5.5 months; HR 0.68; 95% CI 0.48–0.95; *p* = 0.024) [48]. However, the phase II NALIRICC study, enrolling a similar population, did not meet its primary endpoint, demonstrating no benefit in PFS for Nal-IRI + 5-FU/LV vs. 5-FU/LV (median PFS: 2.8 months vs. 2.3 months, respectively), nor did it show a benefit in OS (median OS: 6.9 months vs. 8.2 months, respectively) [49].

There is a role for targeted agents in the management of patients with previously treated advanced BTC, although each of these agents is only indicated for a small fraction of patients with BTC whose tumours harbour specific genomic alterations. Both pemigatinib and infigratinib are inhibitors of the fibroblast growth factor receptor (FGFR) family genes, which are approved by Health Canada for patients with previously treated, unresectable, or metastatic CCA who have detectable FGFR2 gene rearrangements. These approvals were based on two phase II single-arm studies which both reported a median PFS of approximately 7 months in patients with chemo-refractory iCCA and FGFR2 fusions [50,51] (Table 2). The median overall survival was 17.5 months for pemigatinib (median follow-up: 42.9 months) and 12.2 months for infigratinib (median follow-up: 10.6 months) [52,53]. Phase II clinical trials conducted in similar populations have reported median PFS of 8 and 9 months for the FGFR inhibitors derazantinib and futibatinib, respectively [54,55] (Table 2). Phase III trials evaluating first-line therapy with FGFR inhibitors compared with gemcitabine-cisplatin in patients with unresectable or metastatic CCA and FGFR2 fusions are ongoing. These include the FIGHT-302 (pemigatinib, NCT03656536; estimated primary completion: October 2027), and FOENIX-CCA trials (futibatinib, NCT04093362; estimated primary completion: September 2023).

Access to FGFR2 inhibitors in the relapsed setting remains challenging for Canadians with BTC. This is due in part to limited access to biomarker testing, which is required to identify the approximately 10–16% of patients with CCA who harbour FGFR2 fusions [29]. Additionally, pemigatinib, which is the only FGFR2 that continues to be marketed in Canada, is not reimbursed in any province owing to the lack of randomized controlled trials to support its use [61]. This continues to be a barrier to access for all targeted therapies in BTC as clinical trials require enrollment of small genetically defined subpopulations in an already rare disease. Clinical trial enrollment in the second-line setting is particularly difficult as the pool of patients fit to receive subsequent therapy is further decreased.

Activating mutations in isocitrate dehydrogenase 1/2 (IDH1/2) are also targetable in BTC. These mutations are present in 10–20% of patients with CCA. The randomized phase III ClarIDHy trial, which enrolled patients with previously treated CCA and IDH 1/2 mutations, found a statistically significant improvement in PFS for patients receiving the IDH1 inhibitor ivosidenib compared with the placebo (median PFS 2.7 vs. 1.4 months; HR, 0.37; 95% CI 0.25–0.54) [56,57]. A significant difference in OS was not reported in this study, which may be attributed to patient crossover to the experimental arm. Ivosidenib is not available in Canada given the lack of Health Canada approval and special access programs.

Two inhibitors of the neurotrophic tyrosine receptor kinase (NTRK) family proteins, entrectinib and larotrectinib, have a tumour-agnostic indication for patients with unresectable locally advanced or metastatic solid tumors that have confirmed NTRK gene fusions, and no other satisfactory treatment options. Given the low frequency of NTRK fusions in BTC (<1% of patients with CCA), only three patients with CCA receiving TRK inhibitors have been reported in small, single-arm basket studies, two of which achieved partial responses [62,63]. Nevertheless, NTRK inhibitors, which are reimbursed in some Canadian provinces, are a reasonable option for eligible patients with BTC who have otherwise limited options following first-line systemic therapy. Similarly, RET inhibitors have demonstrated efficacy in patients with solid tumours and RET fusions. Although this only represents <2% of patients with cancer, RET inhibitors have demonstrated some activity in heavily pretreated patients with CCA and RET fusions [64,65]. While the RET inhibitor selpercatinib has been approved as a tumour-agnostic therapy by the U.S. Food and Drug Administration, it has not yet been approved in Canada.

Both BRAF and HER2 inhibitors are emerging targeted agents that may be accessible through clinical trial enrollment or special access in patients whose tumours harbour BRAF V600E mutations or HER2 amplification or overexpression, respectively. Dabrafenib and trametinib (BRAF and MEK inhibitors) were investigated in 43 patients with previously treated, unresectable, advanced or metastatic BTC with confirmed BRAF V600E mutations, as part of the phase II ROAR basket trial [58] (Table 2). This regimen achieved an ORR of 51% and median OS of 14 months. The NCI-MATCH trial (subprotocol H) also found a high response rate (75%) for dabrafenib and trametinib in four patients with BRAF V600E-mutated BTC [66]. Clinical trials of anti-HER2 monoclonal antibodies pertuzumab-trastuzumab and trastuzumab deruxtecan have reported ORRs of 23% and 36%, respectively, and median OS results of 10.9 months and 7.1 months in patients with previously treated metastatic BTC [59,60] (Table 2). The phase II BILHER study (NCT03613168) investigating trastuzumab in combination with gemcitabine-cisplatin is complete, but no results have been published yet.

Although individual actionable genomic alterations are present in a small proportion of patients with BTC, collectively, potentially actionable genomic alterations may be present in approximately 30% of BTCs [67,68]. The majority of these alterations are enriched in patients with iCCA, with the exception of HER2 amplification and overexpression which are more common in patients with GBC (10–16%) and eCCA (5–11%) [69]. Given that BTC is genetically diverse, has a dismal prognosis, and has limited treatment options, coupled with fact that the number of therapies being evaluated in clinical trials that target genomic alterations is increasing, next-generation sequencing for patients with advanced and metastatic BTC who are fit to receive systemic therapy is strongly encouraged. Testing early in the disease course, preferably at diagnosis, can allow for treatment planning in the second-line setting. Genomic profiling may be considered in all patients with BTC, including patients undergoing resection, given the high frequency of recurrence. This practice has the potential to improve efficiency and cost-effectiveness and could additionally expand our understanding of BTC.

### 2.4. Discussion on Locoregional Therapy for Palliation

Although systemic therapy is currently the cornerstone of treatment in advanced, unresectable BTC, small, single-centre, retrospective studies have demonstrated impressive survival outcomes with different locoregional therapies, particularly for iCCA [26]. With the lack of prospective, randomized trials, treatment decisions for patients with BTC should be made in consultation with a multidisciplinary team of hepatobiliary surgeons, medical oncologists, radiation oncologists, and interventional radiologists.

Radiofrequency ablation is an option for patients with iCCA whose tumours are <3 cm but are not amenable to surgery. This is based on a meta-analysis evaluating radiofrequency ablation in patients with primary unresectable or recurrent iCCA, where the median OS among the seven studies included ranged from 20 to 60 months, and the pooled 1-, 3-, and 5-year survival rates were 82%, 47%, and 24%, respectively [70]. Stereotactic body radiotherapy (SBRT) has also demonstrated prolonged survival over standard of care for oligometastatic disease in a number of solid tumours [71]. Among the limited retrospective studies of SBRT in BTC, a 2-year OS rate of 41% was observed after SBRT in patients with recurrent BTC following surgery [72] and a median OS of 48 months was reported in patients with small unresectable iCCA receiving SBRT [73]. 

Transarterial chemoembolization (TACE) or radioembolization (TARE) may also be used in patients with unresectable disease. Meta-analyses of studies evaluating TACE and TARE in patients with iCCA have reported pooled median OS values of 15.9 months and 12.7 months, respectively [74,75]. In phase II studies evaluating a combination of TACE or TARE with gemcitabine-cisplatin, median OS values beyond 20 months were reported, with approximately one quarter of patients being downstaged for eligibility for resection or ablation [76,77].

Additionally, radiologists play an important role in managing disease-related complications and symptoms. For symptoms related to disease metastasis, randomized studies have provided evidence for low-dose palliative radiation in improving symptoms and potentially survival for patients with metastatic disease failing systemic therapy [78,79]. Patients with BTC frequently have complications relating to bile duct obstruction, causing jaundice (and related symptoms of diarrhea, sleep disturbances, anorexia, and pruritus), infection, and liver dysfunction. Biliary drainage can relieve these symptoms and may have an impact on survival by allowing patients to be eligible for systemic therapy. In the context of unresectable disease, it may be performed percutaneously or endoscopically (with stenting) depending on institutional experience, anatomical and biological factors, and the presence of cholangitis [80]. Tumour progression can lead to decreased stent patency requiring stent replacement [81]. Several studies have shown prolonged stent patency with the use of local therapies, including intraluminal brachytherapy and radiofrequency ablation (both with and without chemotherapy), leading to improved outcomes in patients [82,83,84,85].

## 3. Adjuvant Therapy for Biliary Tract Cancer

### 3.1. Recommendations

9. Patients with BTC should be considered for adjuvant chemotherapy with capecitabine following curative-intent resection.

### 3.2. Discussion on Adjuvant Therapy

The phase III BILCAP trial is the only study generating positive results showing the benefit of adjuvant therapy [17]. In this study, 447 patients with CCA or muscle-invasive GBC were randomized to adjuvant capecitabine or surveillance following surgery. The per-protocol analysis (*n* = 430) found a statistically significant improvement in OS and disease-free survival (DFS) for the capecitabine arm (median OS: 53.0 months vs. 36.0 months, *p* = 0.028; median DFS: 25.9 months vs. 17.4 months, *p* = 0.0093), although only a numerical difference, not statistical significance, was reached in the intention-to-treat population [17]. Capecitabine is currently the recommended option for adjuvant therapy in Canada. It is typically administered at a dose of 1250 mg/m^2^, twice-daily on days 1–14 for eight 21-day cycles (as per the BILCAP protocol); however, a dose reduction to 1000 mg/m^2^ may be considered at initiation or during treatment based on the anticipated or observed toxicity.

Other phase III studies evaluating different adjuvant chemotherapy regimens, including gemcitabine, gemcitabine-oxaliplatin, and 5-fluorouracil plus folinic acid, have not demonstrated a statistically significant improvement in OS compared with surveillance [18,19,20] (Table 3). Patient heterogeneity, low treatment completion, and underpowered studies may have contributed to the lack of benefit for adjuvant therapy in these trials [86]. The phase III STAMP trial conducted in Asia, evaluating gemcitabine-cisplatin as adjuvant therapy compared with surveillance, also failed to demonstrate a statistically significant improvement in OS for patients with resected eCCA [87]. The ACTICCA-1 trial is currently evaluating adjuvant therapy with gemcitabine-cisplatin compared with capecitabine in a phase III randomized trial across Europe and Australia for patients with BTC who underwent curative-intent resection (NCT02170090), which will confirm whether the current standard of care for metastatic BTC, gemcitabine-cisplatin, is valuable as adjuvant therapy in BTC.

Having positive microscopic margins after resection is known to be a negative prognostic factor for survival in patients with BTC undergoing curative surgery. A reduced benefit for adjuvant therapy in patients with R1 resection was observed in subgroup analyses of some of the aforementioned phase III studies, although none were powered to measure this [17,18,19]. The phase II SWOG S0809 study evaluating adjuvant chemoradiation therapy in patients with eCCA or GBC found that recurrence-free survival and OS were similar between patients with R0 and R1 resections [88]. A systematic review by Horgan et al., found that patients with R1 but not R0 resection benefited from adjuvant radiation therapy [89]. Although this suggests adjuvant chemoradiation may have a benefit in patients with R1 residual disease, these data should be interpreted with caution, and the possibility of improved survival for R1 patients should be further explored in randomized prospective trials.

## 4. Neoadjuvant Therapy for Biliary Tract Cancer

### 4.1. Recommendations

10. There is no randomized data supporting the routine use of neoadjuvant treatment in surgically resectable BTC. However, cases can be reviewed in a multi-disciplinary fashion where downstaging may be warranted in borderline cases.

### 4.2. Discussion on Neoadjuvant Therapy

One potential advantage for neoadjuvant therapy in BTC is improving the R0 resection rate. This was demonstrated in a retrospective study reviewing 1450 patients with stage I-III CCA in the United States National Cancer Database, which found that R0 resection was achieved more frequently in those who received neoadjuvant chemotherapy vs. those who had upfront surgery followed by adjuvant chemotherapy (71.2% vs. 61.6%, *p* = 0.02) [90].

The majority of studies in the neoadjuvant setting have focused on downstaging in patients with unresectable BTC. The largest prospective study yet, evaluating neoadjuvant treatment in patients with locally advanced or borderline resectable GBC (*n* = 160) found that neoadjuvant therapy with gemcitabine-platinum therapy allowed for 41% of patients to proceed to surgery, with an R0 rate of 95% [91]. Among these patients, DFS and OS were 25 months and 49 months, respectively. Chemoradiotherapy may also be useful in the neoadjuvant setting for BTC; however, prospective trials have not been published. A large retrospective study of patients with resectable CCA or GBC compared 27 patients who received neoadjuvant external beam radiation therapy plus gemcitabine with 79 patients who had surgery without neoadjuvant therapy [92]. This study found the 3-year relapse-free survival (RFS) and OS rates to be higher in patients treated with versus without neoadjuvant therapy (RFS: 78% vs. 58%, respectively, *p* = 0.0263; OS: 85% vs. 69%, *p* = 0.00187). Several active phase III trials are exploring different combinations of chemotherapy with radiotherapy, novel agents (lenvatinib), and immunotherapy in the neoadjuvant setting, which will, hopefully, provide guidance on the optimal use of neoadjuvant therapy in BTC.

The use of neoadjuvant chemoradiotherapy followed by liver transplantation is trending upwards, and this practice is now well established for patients with small unresectable, non-metastatic perihilar CCA following protocols similar to those developed by the Mayo clinic. The Mayo clinic protocol includes 45 Gy external-beam radiation therapy (plus 5-fluorouracil as a sensitizer) twice-daily over 3 weeks, followed by endoluminal brachytherapy and concomitant 5-fluoruracil, delivered continuously [93]. The study reporting on this protocol found that among 19 eligible patients, only 60% went on to transplant, with the primary reason for dropping out being the development of metastatic nodal or peritoneal disease after neoadjuvant therapy. In a long-term follow-up of 28 patients who received a transplant, the 5-year survival rate was 82% [94]. Additional single-centre studies using a similar protocol have reported mixed results. A single-centre study based in Ireland found 70% of patients who received chemoradiotherapy proceeded to liver transplantation, and the 5-year survival rate for those patients was 55% [95]. A small study based out of Princess Margaret Cancer Centre and Toronto General Hospital found that only 33% of patients who received chemoradiotherapy proceeded to liver transplantation [23]. The overall 2-year survival and post-transplant 2-year survival were 35.3% and 55.6%, respectively. Together, these studies provide support for the use of neoadjuvant chemotherapy prior to liver transplantation in patients with unresectable perihilar CCA; however, given the variability in drop-out and survival rates, further studies to clarify the optimal selection criteria are needed.

Based on the relative success of liver transplantation in perihilar CCA, its use has more recently been revisited for iCCA despite poor outcomes historically [25,96,97,98] Currently, neoadjuvant systemic therapy followed by liver transplantation in patients with advanced, unresectable iCCA should only be performed under investigational protocols. A small prospective study based out of the University Health Network in Toronto, Canada is underway (NCT04195503). This study may help to clarify the benefit of neoadjuvant systemic therapy and liver transplantation in these patients.

## 5. Summary and Conclusions

**Figure 2:**
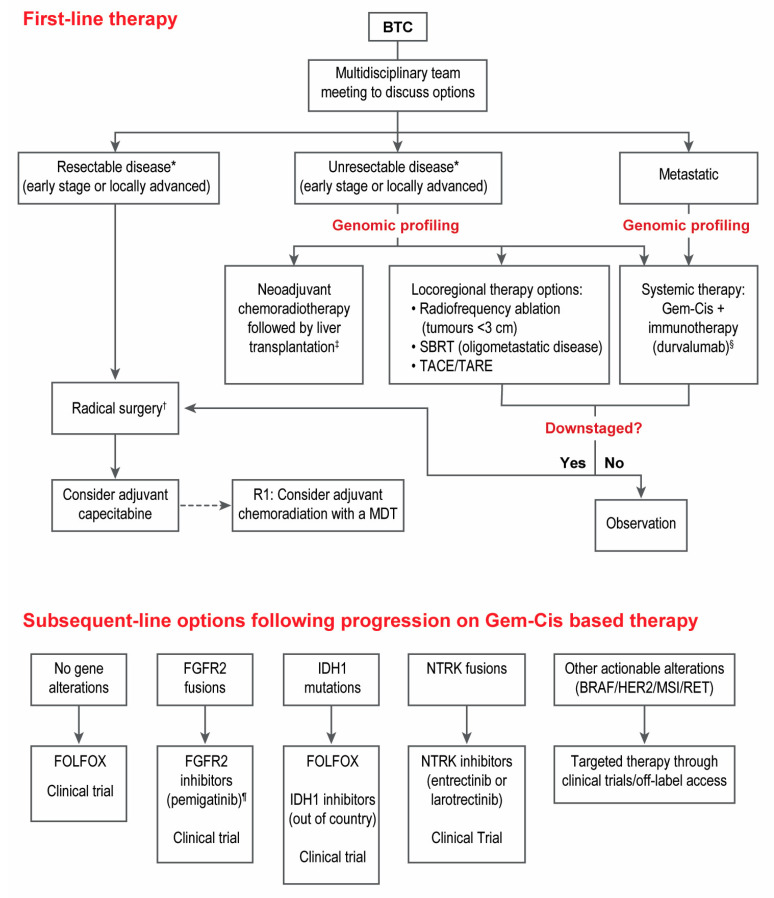
Proposed algorithm for systemic treatment of biliary tract cancer. * Eligibility for resection is based on a number of factors, including presence of distant metastasis, vascular invasion, feasibility of reconstruction, expected volume and function of liver remnant, and performance status. It should be assessed by a hepatobiliary surgeon in consultation with a multidisciplinary team. ^†^ Genomic profiling can be considered using surgical specimen given the high rate of recurrence in these patients. ^‡^ Should be considered in patients with pCCA and remains investigational for iCCA. ^§^ Durvalumab is currently the only immunotherapy agent with a Health Canada indication for treatment of patients with advanced or metastatic BTC. ^¶^ Pemigatinib is currently the only FGFR2 with a Health Canada indication in BTC that is marketed in Canada. BTC, biliary tract cancer; Gem-Cis, gemcitabine-cisplatin; R1, residual disease; SBRT, stereotactic body radiation therapy; TACE/TARE, Transarterial chemoembolization/Transarterial radioembolization.

The landscape of systemic treatment for patients with advanced unresectable or metastatic BTC has evolved, which is reflected in the recent updates of international guidelines [33,45]. Differences in practice and drug access in Canada impact treatment recommendations and algorithms, which are summarised in Table 4 and Figure 2, respectively. After remaining unchanged for two decades, the standard of care for the first-line treatment of advanced unresectable or metastatic BTC now includes the addition of immune checkpoint inhibitors to the chemotherapy backbone of gemcitabine-cisplatin. This was first established with data from the TOPAZ-1 trial showing significantly improved OS for durvalumab plus gemcitabine-cisplatin compared to the placebo plus gemcitabine-cisplatin and was more recently confirmed in the KEYNOTE-966 trial evaluating pembrolizumab plus gemcitabine-cisplatin. However, the latter is not yet available in Canada for patients with BTC.

Biomarker-driven targeted agents continue to be explored in the first-line and relapsed setting. However, they face challenges with approval and reimbursement that can prevent their adoption in practice. This stems from the lack of large phase III randomized trials that are often not feasible to perform in small, genetically defined populations. Lack of molecular testing may also limit the use of targeted agents, but testing is strongly encouraged given the growing number of relevant actionable mutations in BTC. Other effective systemic therapy options remain limited, particularly following a relapse on initial therapy. For this reason, clinical trials should be strongly considered for patients with advanced metastatic BTC.

Management of patients with a locally advanced disease can be particularly challenging, as there are many effective locoregional options but few comparative trials to guide treatment decisions. Heterogeneity in disease distribution and features also make treatment decisions complex, which together highlight the need for multidisciplinary teams to manage these patients. Systemic therapy may be combined with locoregional therapies in the adjuvant and neoadjuvant settings. Currently, the standard of care is to administer adjuvant capecitabine following curative-intent surgery. There is no evidence for the routine use of neoadjuvant therapy in BTC; however, it may be considered in some patients for downstaging.

Despite recent therapeutic advances in BTC, many unmet needs remain. These include the elucidation of reliable biomarkers to predict those patients with a prolonged response to immunotherapy, as well as prospective studies comparing systemic and locoregional therapies in patients with advanced disease. Access to therapies and molecular testing continues to be a barrier to providing optimal care to Canadian patients with BTC. A novel approach to evaluating the value of new therapies and international collaborative efforts for clinical trials of novel therapies, particularly those targeting rare gene alterations, are required to improve care for patients with BTC.

## Figures and Tables

**Table 1:** Key clinical trials comparing new therapy combinations against gemcitabine-cisplatin for the treatment of unresectable advanced or metastatic BTC in the first-line setting.

Trial Name/Phase	Population	Treatment Arms	Response Rate	PFS		OS		
				Median (months	HR (95% CI); *p*-Value	Median (Months	HR (95% CI); *p*-Value	1 or 2-yr Rate
ABC-02 Trial [28] Phase III	Advanced BTC *N* = 410	Arm A: Gem-Cis	A: 26.1%	A: 8.0	0.64 (0.51–0.77); *p* < 0.001	A: 11.7	0.64 (0.52–0.80); *p* < 0.001	Not reported
		Arm B: Gem	B: 15.5%	B: 5.0		B: 8.1		
Sharma et al., 2019 [34] Phase III	Unresectable GBC *N* = 243	Arm A: GEMOX	A: 25.2%	A: 5.0	Not reported; *p* = 0.047	A: 9.0	0.78 (0.60−1.02); *p* = 0.057	A: 32% at 1 yr
		Arm B: Gem-Cis	B: 23.4%	B: 4.0		B: 8.3		B: 24% at 1 yr
PRODIGE 38 [35] Phase II/III	Advanced BTC *N* = 190	Arm A: mFOLFIRI-NOX	A: 25.0%	A: 6.2	0.78 (0.57–1.05); *p* = 0.11	A: 11.7	Not reported	A: 48% at 1 yr
		Arm B: Gem-Cis	B: 19.4%	B: 7.4		B: 13.8		B: 56% at 1 yr
Lee et al., 2014 [36] Phase II	Advanced BTC *N* = 93	Arm A: Cape-Cis	A: 27.3%	A: 5.2	Not reported; *p* = 0.016	A: 10.7	Not reported; *p* = 0.365	Not reported
		Arm B: Gem-Cis	B: 6.1%	B: 3.6		B: 8.6		
Markussen et al., 2020 [37] Phase II	Advanced BTC *N* = 96	Arm A: GEMOX-Cape	A: 17%	A: 5.7	0.721 (not reported); *p* = 0.1	A: 8.7	0.731 (not reported); *p* = 0.1	Not reported
		Arm B: Gem-Cis	B: 16%	B: 7.3		B: 12.0		
SWOG 1815 [38] Phase III	Advanced BTC *N* = 441	Arm A: GAP	A: 34%	A: 8.2	0.92 (0.72–1.16); *p* = 0.47	A: 14	0.93 (0.74–1.19); *p* = 0.58	Not reported
		Arm B: Gem-Cis	B: 25%	B: 6.4		B: 12.7		
TOPAZ-1 [39,40] Phase III	Advanced/unresectable BTC *N* = 685	Arm A: Durva-lumab + Gem-Cis	A: 26.7%	A: 7.2	0.75 (0.63−0.89); *p* = 0.001	A: 12.9	0.76 (0.64−0.91); *p*, not reported	A: 23.6% at 2 yr
		Arm B: Placebo + Gem-Cis	B: 18.7%	B: 5.7		B: 11.3		B: 11.5% at 2 yr
KEYNOTE-966 [41] Phase III	Advanced/unresectable BTC *N* = 1069	Arm A: Pembrolizumab + Gem-Cis	A: 28.7%	A: 6.5	0.86 (0.75–1.00); *p* = 0.023 *	A: 12.7	0.76 (0.64−0.91); *p*, not reported	A: 25% at 2 yr
		Arm B: Placebo + Gem-Cis	B: 28.5%	B: 5.6		B: 10.9		B: 18% at 2 yr

* Not considered statistically significant given the applied significance boundary of *p* = 0.0125. BTC, biliary tract cancer; Cape, capecitabine; Cis, cisplatin; CI, confidence interval; GAP, gemcitabine + cisplatin + albumin-bound paclitaxel; GBC, gall bladder cancer; Gem, gemcitabine; Gem-Cis, gemcitabine + cisplatin; GEMOX, gemcitabine + oxaliplatin; HR, hazard ratio; mFOLFIRINOX, modified FOLFIRINOX (5-fluorouracil + leucovorin + irinotecan + oxaliplatin); N, number; OS, overall survival; PFS, progression-free survival; yr, year.

**Table 2:** Key clinical trials evaluating biomarker-driven targeted agents in biliary tract cancer.

Trial Name/Phase	Population	Treatment Arms	Target/Biomarker	Response Rate	Median PFS (Months)	Median OS (Months)
FIGHT-202 [52] Phase II	Chemotherapy refractory advanced iCCA *N* = 107	Pemigatinib	FGFR 1–3 FGFR2 fusions	37% *	7.0	17.5
Javle et al., 2021 [53] Phase II	Chemotherapy refractory advanced iCCA *N* = 108	Infigratinib	FGFR 1–4 FGFR fusions	23.1% *	7.3	12.2
FIDES-01 [54] Phase II	Chemotherapy refractory advanced iCCA *N* = 103	Derazantinib	FGFR 1–3 FGFR2 fusions	21.4% *	8.0	15.9
FOENIX-CCA2 [55] Phase II	Chemotherapy refractory advanced iCCA *N* = 103	Futibatinib	FGFR 1–4 FGFR fusions	41.7% *	8.9	20.0
ClarIDHy [56,57] Phase III	Advanced/ metastatic CCA Second line *N* = 187	Arm A: Ivosidenib	IDH1 IDH1 mutations	2%	2.7	10.3
		Arm B: Placebo		0%	1.4	7.5
					HR_(ivo vs. plb)_ 0.37 (95% CI 0.25–0.54) *p* < 0.0001	HR_(ivo vs. plb)_ 0.79 (95% CI 0.56–1.12) *p* = 0.09
ROAR [58] Phase II	Advanced/metastatic CCA Second line *N* = 43	Dabrafenib + trametinib	BRAF + MEK BRAF V600E	51%	9	14
MyPathway [59] Phase II	Previously treated metastatic BTC *N* = 39	Pertuzumab + trastuzumab	HER2 HER2 amplification/overexpression	23%	4	10.9
HERB [60] Phase II	Previously treated metastatic BTC *N* = 22	Trastuzumab deruxtecan	HER2 HER2 amplification/overexpression	36.4%	4.4	7.1

* Response rate included for FGFR2 fusions/rearrangements only. BTC, biliary tract cancer; CI, confidence interval; CCA, cholangiocarcinoma; HR, hazard ratio; iCCA, intrahepatic cholangiocarcinoma; ivo, ivosidenib; m, median; OS, overall survival; PFS, progression-free survival; plb, placebo.

**Table 3:** Key clinical trials evaluating adjuvant chemotherapy following curative-intent resection in biliary tract cancer.

Trial Name/Phase	Population	Treatment Arms	Median RFS/DFS (Months)	HR for RFS/DFS (95% CI); *p*-Value	Median OS (Months)	HR for OS (95% CI); *p*-Value
ESPAC-3 trial [18] Phase III	eCCA, AC *N* = 428	Arm A: 5FU + FA	A: 23.0	HR _A vs. C_ 0.69 (0.51–0.95); *p* = 0.02 HR _B vs. C_ 0.68 (0.50−0.95); *p* = 0.02	A: 38.9	HR _A vs. C_ 0.95; (0.71–1.28); *p* = 0.74 HR _B vs. C_ 0.77 (0.57−1.05); *p* = 0.10
		Arm B: Gem	B: 29.1		B: 45.7	
		Arm C: Surgery alone	C: 19.5		C: 35.2	
BCAT trial [19] Phase III	eCCA *N* = 225	Arm A: Gem	A: 36.0	0.93 (0.66−1.32); *p* = 0.693	A: 62.3	1.01 (0.70−1.45); *p* = 0.964
		Arm B: Surgery alone	B: 39.9		B: 63.8	
PRODIGE 12-ACCORD 18 [20] Phase III	iCCA, eCCA GBC *N* = 194	Arm A: GEMOX	A: 30.4	0.88 (0.62−1.25); *p* = 0.48	A: 75.8	1.08 (0.70−1.66); *p* = 0.74
		Arm B: Surgery alone	B: 18.5		B: 50.8	
BILCAP [17] * Phase III	iCCA, eCCA GBC *N* = 447	Arm A: Cape	A: 25.9	0.70 (0.54–0.92); *p* = 0.0093	A: 53.0	0.75 (0.58–0.97); *p* = 0.028
		Arm B: Surgery alone	B: 17.4		B: 36.0	
STAMP [87] Phase II	eCCA *N* = 101	Arm A: Gem-Cis	A: 14.3	0.96 (0.71–1.30); *p* = 0.86	A: 35.7	1.08 (0.72–1.64); *p* = 0.81
		Arm B: Cape	B: 11.1		B: 35.7	

* Data reported from the per-protocol analysis of 430 patients. AC, ampullary cancer; Cape, capecitabine; CCA, cholangiocarcinoma; Cis, cisplatin; eCCA, extrahepatic cholangiocarcinoma; FA, folinic acid; 5FU, 5-fluorouracil; GBC, gallbladder cancer; Gem; gemcitabine, GEMOX; gemcitabine + oxaliplatin; iCCA, intrahepatic cholangiocarcinoma; N, number; OS, overall survival; RFS, recurrence-free survival.

**Table 4:** Summary of Recommendations.

**Recommendation**	Level of Evidence *
Systemic therapy for unresectable advanced or metastatic BTC	
Patients with advanced unresectable or metastatic BTC should be considered for first-line treatment with gemcitabine-cisplatin plus immunotherapy (durvalumab).	I
2. Genomic profiling of relevant BTC genes by next-generation sequencing is strongly suggested for all patients with advanced unresectable or metastatic BTC that are fit to receive systemic therapy. Profiling is preferred at diagnosis to allow for treatment planning and access to targeted agents in the second line.	V
3. FOLFOX should be considered in the second-line setting after progressing on gemcitabine-cisplatin-based therapy for patients with advanced BTC with no actionable genomic alterations.	I
4. Patients with CCA who harbour FGFR2 fusions should be considered for treatment with FGFR2 inhibitors (pemigatinib) after progressing on one prior line of systemic therapy.	III
5. Patients with CCA who harbour IDH1 mutations do not have access to IDH1 inhibitors in Canada. Alternative means of access may be considered but is challenging.	I
6. Patients with BTC who harbour NTRK fusions should be considered for treatment with NTRK inhibitors (entrectinib or larotrectinib) after progressing on one prior line of systemic therapy.	III
7. Patients with BTC who harbour other actionable genomic alterations (e.g., BRAF, HER2, RET, MSI) should be considered for targeted therapy through clinical trials or other means of access.	V
8. Locoregional therapies for palliation should be considered and discussed with multidisciplinary teams.	V
Adjuvant therapy	
9. Patients with BTC should be considered for adjuvant chemotherapy with capecitabine following curative-intent resection.	II
Neoadjuvant therapy	
10. There is no randomized data supporting the routine use of neoadjuvant treatment in surgically resectable BTC. However, cases can be reviewed in a multidisciplinary fashion where downstaging may be warranted in borderline cases.	V

* Levels of evidence were based on definitions from the European Society for Medical Oncology clinical practice guideline for diagnosis, treatment and follow-up of biliary tract cancer [33]. These include: Level 1: Evidence from at least one large randomised, controlled trial of good methodological quality (low potential for bias) or meta-analyses of well-conducted randomised trials without heterogeneity; Level II: Small randomised trials or large randomised trials with a suspicion of bias (lower methodological quality) or meta-analyses of such trials or of trials with demonstrated heterogeneity; Level III: Prospective cohort studies; Level IV: Retrospective cohort studies or case-control studies; Level V: Studies without control group, case reports, expert opinions. BTC, biliary tract cancer; CCA, cholangiocarcinoma.

## Data Availability

Not applicable.
